# Molecular Insights into Fluoride Ion Uptake and Selectivity in the CLCF Fluoride/Proton Antiporter

**DOI:** 10.1021/acs.jpcb.4c08174

**Published:** 2025-04-15

**Authors:** Akihiro Y. Nakamura, Takuya Mabuchi

**Affiliations:** † Graduate School of Engineering, Tohoku University, 2-1-1, Katahira, Aoba-ku, Sendai 980-8577, Japan; ‡ Institute of Fluid Science, Tohoku University, 2-1-1, Katahira, Aoba-ku, Sendai 980-8577, Japan

## Abstract

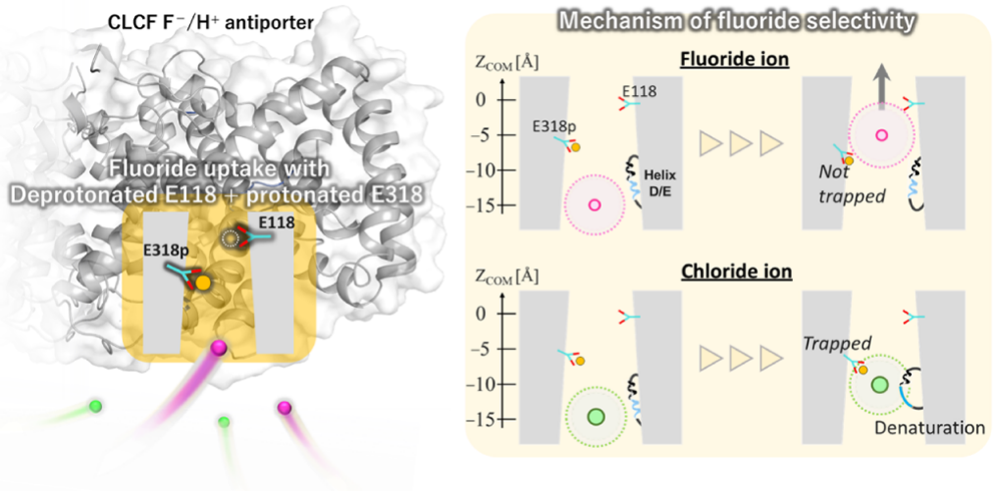

In this study, we investigated the effect of the protonation state of glutamate E118 (Gluex) and glutamate E318 (Gluin) on fluoride ion uptake and selectivity in the CLCF F^–^/H^+^ antiporter using molecular dynamics simulations. Analyses of pore size and the potential of mean force (PMF) revealed that fluoride uptake is facilitated under the deprotonated E118 and protonated E318 state, consistent with the fluoride uptake state proposed in the original windmill mechanism. In this state, an increased pore size reduces the energy barrier, promoting fluoride transport from the intracellular solution to the intracellular binding site (S_cen_). Interestingly, we also observed a helix-to-coil transition (residues 74–87) in the presence of chloride at S_cen_, which enhances chloride dehydration and stabilizes its interaction with the coil structure. This conformational change likely impedes chloride transport, contributing to fluoride ion selectivity. Our findings confirm that fluoride ion selectivity is enhanced in the E118_E318p state, reinforcing its role in the original windmill mechanism. Additionally, we propose that refining the fluoride uptake process in the modified windmill mechanism could lead to a comparable selectivity mechanism, ultimately converging on a unified fluoride-selective uptake mechanism that integrates key aspects of both pathways.

## 1 Introduction

Fluoride ions at concentrations as low as 10–100 μM are known to sufficiently inhibit the action of enolase and phosphotransferase enzymes in bacterial cells,^1,2^ and many bacteria (unicellular organisms) use membrane proteins to expel fluoride ions.^3^ Representative proteins include ion channels that specifically transport fluoride ions (i.e., fluoride channels)^4−6^ and transporters belonging to the chloride channel (CLC) superfamily^7−9^ of anion channels/transporters, such as the fluoride/proton antiporter of the fluoride-specialized variant of the CLC family (CLCF F^–^/H^+^ antiporter).^10,11^

The CLCF F^–^/H^+^ antiporter is similar to the homolog of the CLC Cl^–^/H^+^ antiporter from *E. coli* (CLC–ec1).^12^ A glutamate residue, E118 (Gluex), in the CLCF F^–^/H^+^ antiporter^13^ is in a similar position to the external gating glutamate residue, E148 in CLC–ec1.^14,15^ Each CLCF F^–^/H^+^ antiporter monomer has two fluoride ion binding sites located at the canonical chloride ion binding sites, as in CLC–ec1: one situated near the pore exit (S_ext_) and one situated at the center (S_cen_). On the other hand, there are differences in the selectivity for fluoride ions over chloride ions and the stoichiometric exchange of two chloride ions for one H^+^ with CLC–ec1. Furthermore, in the CLCF F^–^/H^+^ antiporter, the methionine residue (M79) is situated at the position corresponding to the strictly conserved chloride-coordinating serine (S107), which exhibits chloride ion selectivity in CLC-ec1.^16^ In addition, there is an extra glutamate (E318) near its M79, a characteristic that is absent in the CLC.

Miller et al.^10^ have proposed the original windmill transport model to elucidate the 1:1 fluoride/proton stoichiometry based on experimental ion flux measurements and crystal structure analyses. This mechanism describes the uptake and export of fluoride ions through the CLCF F^–^/H^+^ antiporter, coordinated by protonation and deprotonation of E118. Specifically, when E118 is protonated by the extracellular solution, both anion binding sites (S_cen_ and S_ext_) are occupied with fluoride ions. As E118 rotates toward the intracellular solution and releases its proton, the fluoride ion in S_cen_ is also released intracellularly, allowing the anionic side chain of E118 to occupy the vacant binding site. Then, E118 continues its rotation, exporting the remaining fluoride ion into the extracellular solution, while a new fluoride ion enters S_cen_, completing the transport cycle. This proposed mechanism highlights several key features: 1) Fluoride uptake occurs when both binding sites are occupied by fluoride ions; 2) E118 can directly interact with both the extra- and intracellular solutions to accept and release the transported proton; and 3) E118 plays a similar role as E148 in the chloride ion-transporting CLCs. Several computational studies have been conducted to investigate this mechanism. Chon et al.^17^ examined anion pathways in both wild-type (WT) and mutant forms (E118Q and V319G), revealing p*K*_a_ shifts for glutamate and aspartate residues, which indicate that E118 is readily protonated when fluoride occupies its binding site. Additionally, pore size analysis demonstrated different structural states: WT and E118Q exhibited inward–open–outward–occluded structures, whereas V319G showed inward–closed–outward–occluded. Carloni et al.^18^ proposed a proton release mechanism with a 1:1 stoichiometry using QM/MM simulations. In their model, when E118 and E318 are both protonated, E118 rotates without an energy barrier, and the proton is eventually released into the intracellular solution as hydrofluoric acid. Chon and Lin’s study^19^ further deepened the understanding of the original windmill mechanism by performing QM/MM simulations, demonstrating that E118 expels the fluoride ion from S_ext_, followed by the reoccupation of S_ext_ by an incoming fluoride ion. Their study also suggested that protonated E318 contributes to the recruitment of fluoride ions and that hydrated fluoride ions do not cause lockdown in CLCF. Mills et al.^20^ proposed the modified windmill mechanism, which differs from the original model. Their molecular dynamics (MD) simulations suggested that only a single fluoride ion is present in the transport pathway at a time, rather than two as suggested by Miller et al.^10^ Free-energy calculations indicated that when S_ext_ is unoccupied, protonated E118 rotates toward the intracellular solution, facilitating fluoride uptake and proton export. This mechanism redefines the role of E118, proposing an alternative transport cycle.

However, while both the original and modified windmill mechanisms provide insights into the overall fluoride uptake process, neither fully addresses the molecular basis of fluoride ion selectivity over chloride ions. The specific factors that govern ion discrimination in the CLCF F^–^/H^+^ antiporter remain unclear. In this study, we used MD simulations to investigate the effects of the protonation states of glutamate residues E118 (Gluex) and E318 (Gluin) on fluoride ion uptake and selectivity in the CLCF F^–^/H^+^ antiporter. Our simulations aim to clarify how these protonation states influence not only the transport process but also the mechanisms underlying fluoride ion selectivity.

## 2 Computational Details

The setup involved the CLCF F^–^/H^+^ antiporter (PDB: 6D0J) comprising two symmetrical subunits integrated into a 1-palmitoyl-2-oleoyl-*sn*-glycero-3-phosphoethanolamine (POPE) bilayer using the CHARMM-GUI.^21−23^ The system was immersed in a 150 mM NaCl solution, resulting in a box of 130 Å × 90 Å × 84 Å with approximately 100,000 atoms. For the CLCF F^–^/H^+^ antiporter, we constructed two systems representing the protonation states associated with fluoride uptake: (1) E118_E318p, corresponding to the original windmill mechanism,^10,18,19^ where E118 is deprotonated and E318 is protonated; and (2) E118p_E318, corresponding to the modified windmill mechanism,^20^ where E118 is protonated and E318 is deprotonated. All simulations were conducted using the large-scale atomic/molecular massively parallel simulator (LAMMPS),^24,25^ employing the CHARMM36m force field.^26^ Electrostatic interactions were computed using the particle mesh Ewald method.^27^ Following energy minimization, six stages of equilibration (preequilibrium) were performed, adhering to positional constraints (protein and lipids) according to the CHARMM-GUI protocol.^23,28^ Subsequently, all systems were equilibrated for 200 ns, followed by a production run in the NPT ensemble at 310 K and 1.0 atm pressure for 300 ns with a 2.0 fs time step. For analyses, the potential of mean force (PMF) profiles of anions through the transport pathways were computed from umbrella sampling using the reaction coordinates for each anion. The reaction coordinates were determined by tracking the anion’s movement along the pore in an upward direction, as obtained through constant velocity-steered MD (CV–SMD) simulations.^29^ Prior to the CV-SMD simulations, an anion was positioned and anchored at the intracellular side at *Z*_COM_ = −35.0 Å, where *Z*_COM_ represents the center-of-mass (COM) coordinate relative to the CLCF protein along the membrane normal (*Z*-axis). The system was then re-equilibrated for 2.0 ns. During the CV-SMD simulations, the anion was pulled through the pore toward the extracellular side from *Z*_COM_ = −35.0 to 35.0 Å at a steering speed of 1.0 Å/ns along the + *Z* direction, with a spring force constant of 10 kcal/mol/Å^2^. The snapshots of reaction coordinates for each anion, obtained from SMD simulations, are shown in Figure S1. Following the CV-SMD simulations, umbrella sampling simulations were performed with windows spaced 1.0 Å apart along the reaction coordinates obtained from the SMD simulations. Each window underwent a 2.0 ns equilibration, followed by a 5.0 ns production run using a restraint force of 10 kcal/mol/Å^2^. The PMF profiles were then extracted using the weighted histogram analysis method (WHAM).^30,31^ For the HOLE analysis,^32^ the time-averaged pore radius and its standard deviation along *Z*_COM_ were calculated using 5000 frames collected every 10 ps from the 50 ns of the production run. The distance between E118 and T320 was analyzed using the COM of their side chains to assess structural variations under different protonation states. The coordination number of the central anion was defined as the number of oxygen atoms of water molecules within a 3.0 Å radius. The interaction energy between the anion and protein was calculated as the sum of the electrostatic and Lennard-Jones energies for each residue. The coordination number and interaction energy were obtained across all windows of the umbrella sampling simulations. The root-mean-square fluctuations (RMSF) and secondary structure analyses were performed using the trajectory from the umbrella sampling simulations at a window *Z*_COM_ = −7.5 Å, where an energy dip was found in the PMF analysis. RMSF values were calculated for the Cα atoms of each residue. The secondary structure ratio of each residue was determined using the DSSP algorithm,^33^ and the values were averaged over time in the equilibrated state.

## 3 Results and Discussion

### 3.1 Fluoride Ion Uptake with Different Protonation States

**Figure 1:**
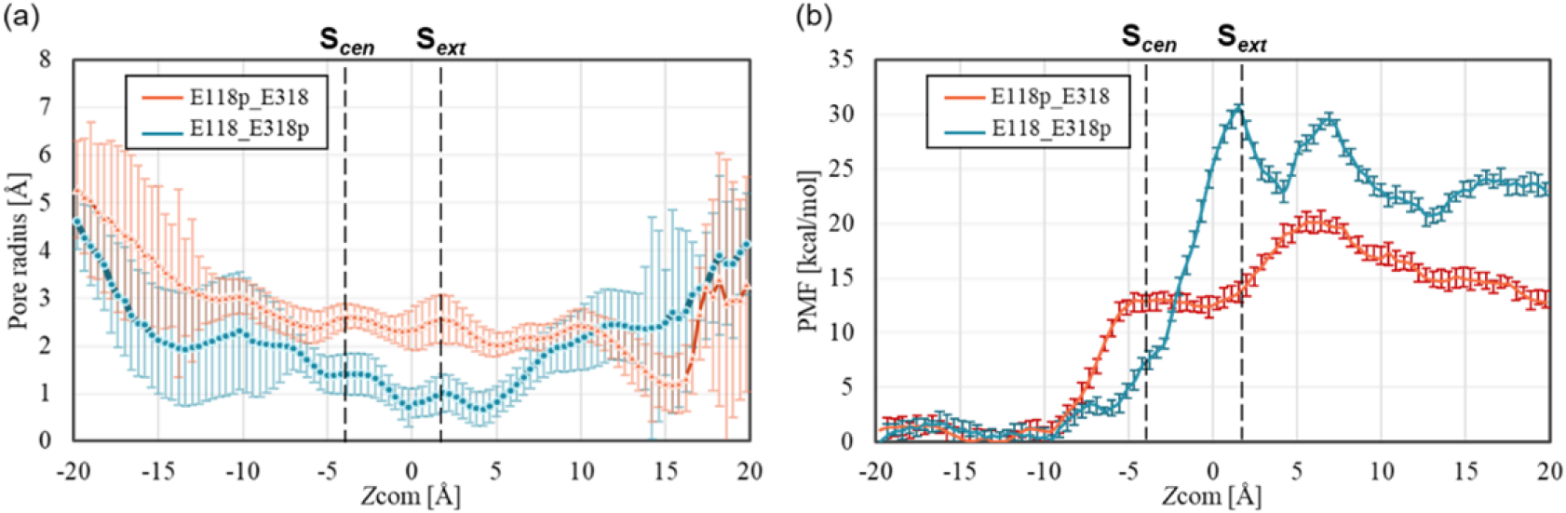
(a) Pore radii for different protonation states of E118 and E318. The line and error bars represent the time-averaged pore radius and its standard deviation along *Z*_COM_, respectively. (b) PMF results on the fluoride ion along *Z*_COM_.

Figure 1a shows the results of the time-averaged pore radius along the *Z*_COM_. For fluoride ion uptake, the results indicate that in both protonation states the pore radius remains larger than the fluoride ion radius of ∼1.2 Å at S_cen_ (*Z*_COM_ = −4.7 Å), ensuring unimpeded fluoride ion uptake. Beyond this point, as the pore expands toward the intracellular region (*Z*_COM_ ≤ −4.7 Å), fluoride ions can pass through without steric hindrance to reach S_cen_. Figure 1b shows the PMF profiles along *Z*_COM_ for fluoride ions with the E118p_E318 and E118_E318p states. From a free energy perspective, the energy barriers from the intracellular solution to S_cen_ are ∼12 kcal/mol for E118p_E318 and ∼7 kcal/mol for E118_E318p, indicating that fluoride ion access is more favorable in the E118_E318p state despite its smaller pore radius compared to E118p_E318. These results suggest that electrostatic interactions play a key role in fluoride ion entry, as the deprotonated E318 in the E118p_E318 state is expected to be more electrostatically repulsive against fluoride ions than the protonated state. This finding is consistent with QM/MM simulations by Chon et al.,^19^ supporting the role of electrostatics in facilitating fluoride uptake in the original windmill mechanism. However, our PMF results differ from those of a previous MD study by Mills et al.,^20^ which reported an opposite trend in energy barriers and consequently proposed the E118p_E318 state as the fluoride ion uptake configuration in the modified windmill mechanism. This discrepancy may be attributed to differences in the methods used to determine the reaction coordinates. Specifically, we used CV-SMD simulations to dynamically generate the reaction coordinates, capturing the transport pathway under nonequilibrium conditions, whereas Mills et al.^20^ employed the tunnel cluster approach, which is a static, equilibrium-based perspective.

Furthermore, while one might expect local minima in the PMF profiles near binding sites S_cen_ and S_ext_, our simulations did not show clear energy wells at either site. This outcome is consistent with Mills et al.’s MD results but contrasts with the QM/MM calculations by Chon et al.,^19^ where a shallow local minimum (∼2 kcal/mol) was observed at Sext. This discrepancy may arise from Chon et al.’s explicit modeling of protonation-state transitions (e.g., E118_E318p to E118p_E318) near S_ext_, which may have transiently stabilized fluoride at S_ext_. The absence of such transitions in our classical MD simulations—where each protonation state is treated independently—may account for the lack of distinct minima. Although the overall PMF trends—an increase before S_ext_ for E118_E318p and after S_ext_ for E118p_E318—are consistent with QM/MM results, these differences highlight the need for future simulations incorporating protonation dynamics in a QM/MM framework. Additionally, similar refinements will be necessary to better understand fluoride stability at S_cen_, thereby providing a better picture of the free energy landscape governing fluoride transport in CLCF.

**Figure 2:**
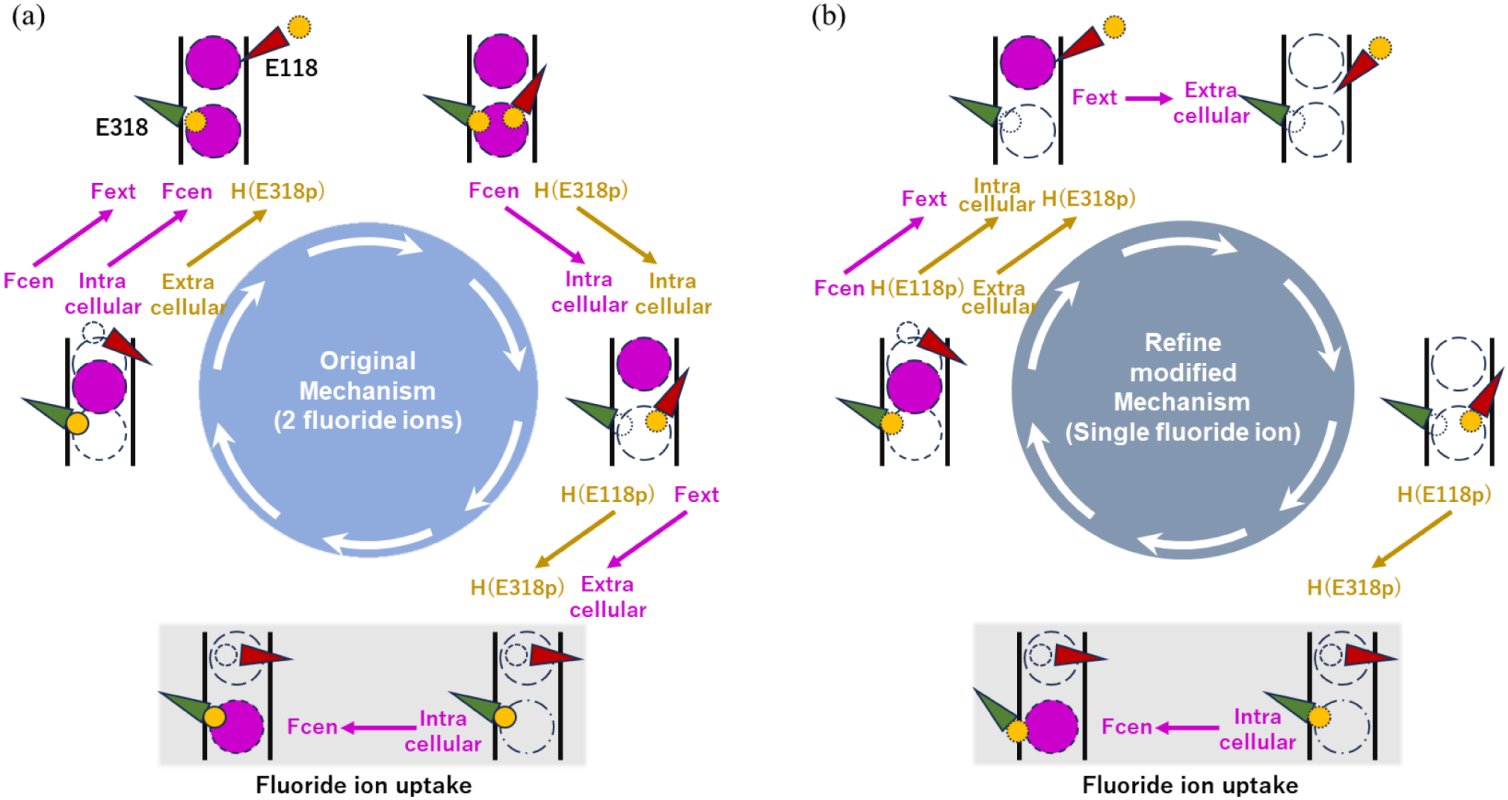
Transport mechanism of the CLCF F^–^/H^+^ antiporter. (a) Original windmill mechanism. (b) Refine version of the modified windmill mechanism. (Fluoride ions are represented as magenta spheres, protons as yellow spheres, E118 as a red triangle, and E318 as a green triangle.)

Given that our results indicate the E118_E318p state is more favorable for fluoride uptake, we propose that our findings provide an opportunity to refine the fluoride uptake process in the modified windmill mechanism by incorporating the E118_E318p state, similar to the original mechanism,^10,18,19^ as shown in Figure 2. Additionally, our simulations showed that two fluoride ions could not coexist stably in the transport pathway (Figure S2), further supporting the idea that fluoride uptake occurs via a single-ion mechanism. While the crystal structure of CLCF shows fluoride occupancy at both the S_cen_ and S_ext_ binding sites, the electron density is not equally distributed between the two, suggesting different binding affinities and not necessarily simultaneous stable occupancy. However, the absence of simultaneous fluoride occupancy at S_cen_ and S_ext_ in our classical MD simulations may also stem from the lack of protonation-state transitions.

For fluoride export at S_ext_, our pore radius analysis in Figure 1a and distance analysis (Figure S3) showed a larger opening under the E118p_E318 state, which is consistent with previous findings reported by Mills et al.^20^ In the initial crystal structure, E118 is oriented in the up position, and our dihedral angle analysis (Figure S4) confirmed that E118 remained stable in this conformation throughout the simulations under both protonation states. While these findings provide support for refining the modified windmill mechanism, we emphasize that the protonation state required for fluoride uptake—specifically, E118 deprotonated and E318 protonated—is consistent across both the original and refined mechanisms. This suggests that both interpretations are viable representations of fluoride uptake in CLCF, ultimately converging on a common fluoride-selective uptake mechanism, as highlighted in this manuscript.

### 3.2 Selective Mechanism of Fluoride Ions in CLCF

**Figure 3:**
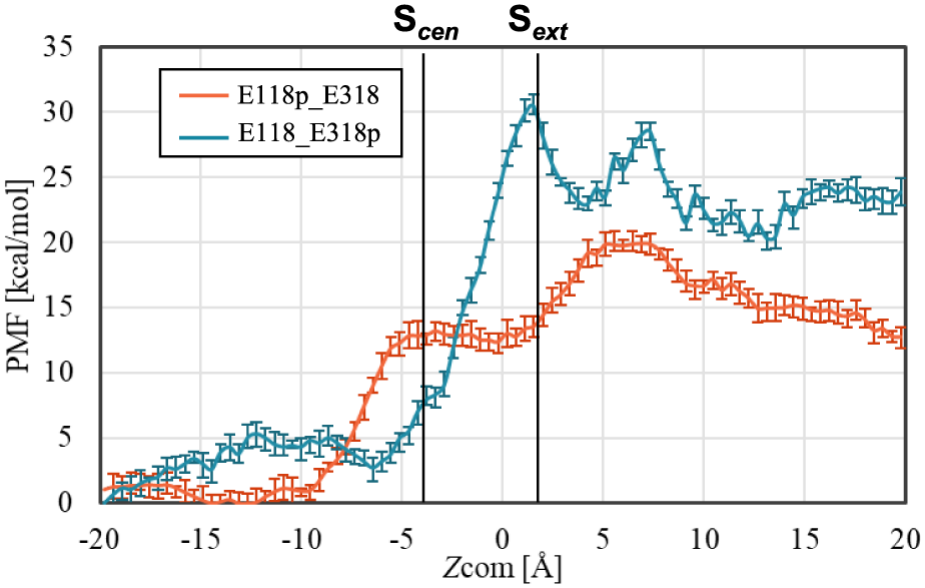
PMF profiles along Z_COM_ for chloride ions in different protonation states.

To investigate the selective mechanism of fluoride ion uptake, the PMF profiles for chloride ions were calculated under different protonation states and compared with those for fluoride ions. Figure 3 shows the PMF profiles along *Z*_COM_ for chloride ions. When fluoride and chloride ions are compared (Figures 1b and 3), the overall PMF trends are similar under the E118p_E318 state. However, under the E118_E318p condition, which corresponds to the fluoride uptake state, a distinct difference emerges in the region at ZCOM < −7.5 Å: chloride exhibits a clear energy barrier that is not observed for fluoride. This suggests that anion selectivity arises specifically at the intracellular side when E118 is deprotonated, reinforcing its essential role in facilitating fluoride ion uptake while restricting chloride transport. This finding highlights that the deprotonated E118 state (E118_E318p) serves as a key condition for fluoride ion uptake in both the original and refined windmill mechanisms, ensuring consistency in the transport process.

**Figure 4:**
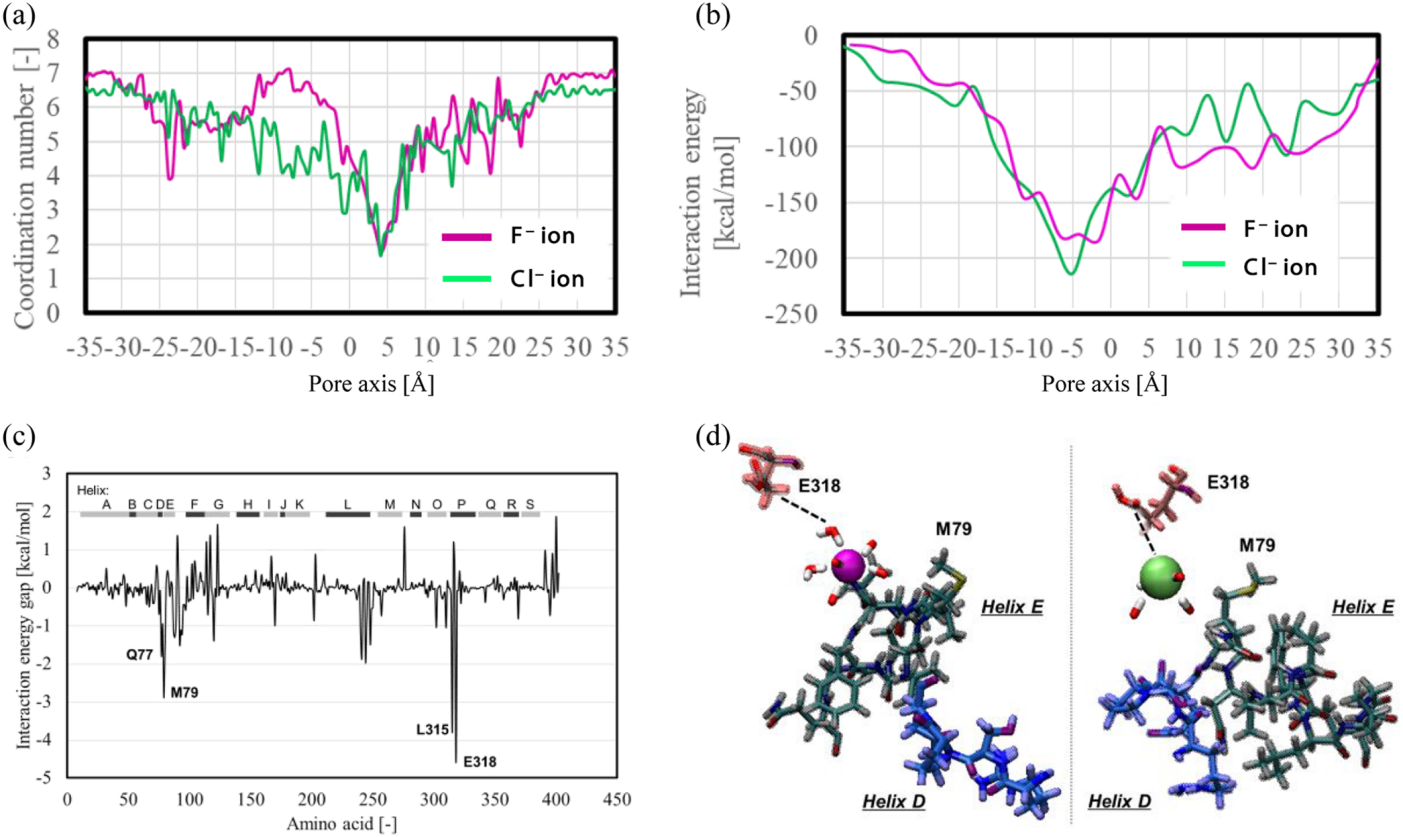
(a) Anion coordination number in the E118_E318p system. (b) Interaction energy between the anion and CLCF F^–^/H^+^ antiporter. (c) Interaction energy gap of each residue between fluoride ion and chloride ion at *Z*_COM_ = −7.5 Å. (d) Two snapshots of the representative frame from the umbrella sampling simulations. Helix D is shown in blue, and Helix E is shown in gray. The anions are shown as spheres (Fluoride ion in magenta and Chloride ion in green). The water molecules are shown as licorice.

To understand anion selectivity in the region of *Z*_COM_ < −7.5 Å under the E118_E318p condition, we analyzed the local ionic structures, including anion hydration and radial distribution functions (RDFs) between the anion and protein residues. Figure 4a shows the number of hydrated water molecules for each anion along the *Z*_COM_ in the E118_E318p system. It should be noted that although the hydration of both chloride and fluoride ions eventually becomes comparable at *Z*_COM_ = ∼5 Å, our analyses focus on the region of *Z*_COM_ < −7.5 Å in this study, as the E118_E318p condition is a key part of the fluoride ion uptake process, while fluoride export occurs under different protonation states at *Z*_COM_ = ∼5 Å in both the original and refined windmill mechanisms. At *Z*_COM_ < −7.5 Å, the number of hydrated water molecules for chloride ions was lower than that for fluoride ions, indicating preferential desolvation of chloride ions. Given that the solvation energy of chloride (∼81 kcal/mol) is lower than that of fluoride (∼111 kcal/mol) in bulk water,^34^ chloride ions are more prone to dehydration in this region. However, hydration alone does not fully explain anion selectivity, as both anions are also stabilized through interactions with pore residues. To further investigate the role of protein residues in anion selectivity, we calculated the RDFs between the anions and surrounding residues at *Z*_COM_ = −7.5 Å (Figure S5). The RDF results show that E318p directly binds to chloride ions, whereas no residues were bound to fluoride ions, indicating that chloride ions undergo dehydration due to binding with E318p.

In addition to direct interactions with E318p, we examined the contributions of neighboring residues to chloride ion binding. The interaction energy between the anions and the CLCF protein along *Z*_COM_ was calculated (Figure 4b). At *Z*_COM_ = −7.5 Å, the interaction energy for chloride ions was found to be about 30 kcal/mol lower than that for fluoride ions, indicating a stronger interaction between chloride ions and the protein. Furthermore, we decomposed the total interaction energy gap between fluoride and chloride into residue-specific contributions at *Z*_COM_ = −7.5 Å (Figure 4c). A negative value indicates that the residue interacts more strongly with chloride ions than with fluoride ions. Notably, E318 and M79, located in helices P and E, respectively, exhibited large negative values, highlighting their contribution to chloride ion stabilization. These results suggest that chloride selectivity at *Z*_COM_ = −7.5 Å is driven not only by direct binding to E318p but also by interactions with M79. This is consistent with the sharp peak of the M79 backbone around the chloride solvation shell observed in the RDF results (Figure S5). Therefore, the binding of chloride ions to E318p triggers dehydration, which subsequently enhances interactions with the backbone of M79 in Helix E, further stabilizing chloride at *Z*_COM_ = −7.5 Å (Figure 4d).

**Figure 5:**
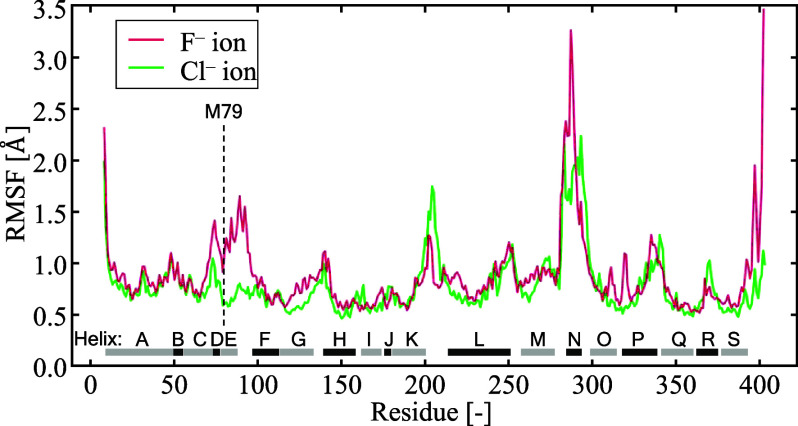
RMSF for the Cα atoms of each residue with an anion located at *Z*_COM_ = – 7.5Å.

To investigate the behavior of residues while interacting with anions, we analyzed the mobility of each residue using the RMSF. Figure 5 shows the RMSF for each residue when the anion was located at *Z*_COM_ = −7.5 Å under the E118_E318p condition, where M79 and E318p were found to strongly interact with the chloride ion, as shown in Figure 4. Although residues around M79 exhibited larger RMSF values for fluoride ions than for chloride ions, the residues around E318 showed no significant differences between the ion types. This suggests that the mobility of the residues around M79 was suppressed due to their strong interactions with chloride ions. While we acknowledge that Helix N exhibits larger RMSF differences between chloride and fluoride simulations, it is not discussed here because it is spatially distant from the Helix D/E region (Figure S6a) and inherently contains coil structures, leading to naturally higher fluctuations. This trend is also observed in the equilibrium RMSF data without anions (Figure S6b), suggesting that its fluctuations are not directly related to anion interactions. Therefore, we focus on Helix D/E, where anion-induced structural changes are more relevant to the transport mechanism.

**Figure 6:**
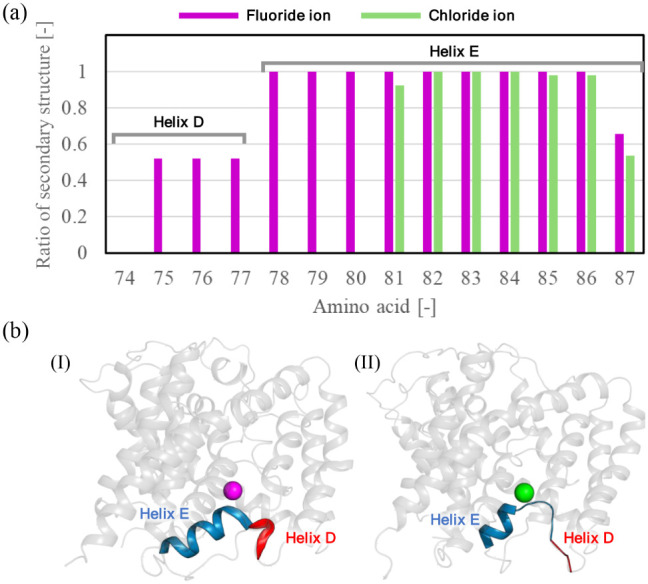
(a) Secondary structure ratio of amino acids in Helix D and Helix E with an anion located at *Z*_COM_ = −7.5Å. (b) Representative snapshots for each anion illustrating the helix–coil transition associated with chloride binding. Helix D is colored red, and Helix E is shown in blue. The anions are shown as spheres (fluoride ion in magenta and chloride ion in green).

**Figure 7:**
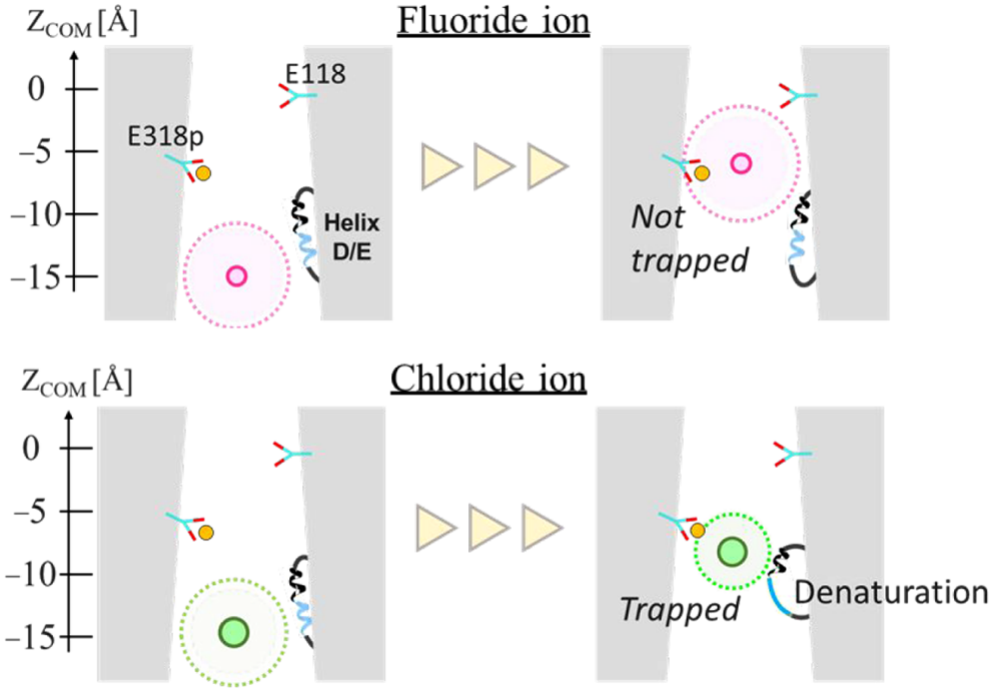
Behavior of the anion in the CLCF F^–^/H^+^ antiporter pathway. (black/lite blue rope: a structure of 74–94 amino acids, straight line circle: anion radius, dashed line circle: hydration radius, and yellow spheres: “protonated”).

Furthermore, the time-averaged fraction of the helix content in Helix D and Helix E was calculated (Figure 6a). The region spanning amino acids 74–80, including M79, underwent a helix-to-coil transition in the presence of chloride ions at *Z*_COM_ = −7.5 Å. Although the 74–77 region in Helix D showed a lower helix fraction (∼55%) even with fluoride ions, this can be attributed to the fragile nature of Helix D based on the crystal structure analysis^10^ as well as our secondary structure analyses without anions (Figure S7). Our results suggest that the binding of chloride to E318p triggers dehydration, which subsequently strengthens interactions with M79. This interaction leads to a helix-to-coil transition, as visualized in Figure 6b. The destabilization of the helical structure enhances chloride binding via backbone interactions, effectively trapping chloride ions and preventing their movement toward S_cen_. In contrast, fluoride ions interact weakly with these residues due to their hydration, allowing the helix to maintain its secondary structure and mobility. These results highlight the critical role of the helix-to-coil transition around M79 and surrounding residues in modulating ion transport and selectivity (Figure 7).

## 4 Conclusions

Using molecular dynamics simulations, we have explored the mechanisms of fluoride ion uptake and selectivity in the CLCF F^–^/H^+^ antiporter, emphasizing the roles of protonation states, hydration structure, and helix stability. Our findings reveal that the E118_E318p state facilitates fluoride uptake, reinforcing its role in the original windmill mechanism. Additionally, we propose that refining the fluoride uptake process in the modified windmill mechanism could lead to a comparable selectivity mechanism, ultimately converging on a unified fluoride-selective uptake mechanism that integrates key aspects of both pathways. Our results suggest that chloride binding to E318p triggers dehydration, which subsequently strengthens interactions with M79. The helix-to-coil transition in residues 74–87, including M79, occurs in the presence of chloride at the binding site S_cen_. This structural transition reinforces chloride trapping and prevents its further transport. In contrast, fluoride ions maintain their hydration shell and exhibit weaker interactions with the protein, preserving the helical structure and allowing for smooth transport toward S_cen_. These results suggest that the transition between the helix and loop structure in the M79-containing region is a critical determinant of fluoride ion selectivity, contributing to the unique transport properties of the CLCF F^–^/H^+^ antiporter.

## Supporting Information Available

Reaction coordinates of ions in SMD simulations; system equilibration with two fluoride ions; distance analysis between key residues; dihedral angle dynamics of E118; RDFs between protein residues and water molecules around anions; helix locations and residue-wise RMSF; and secondary structure analysis of Helices D and E (PDF)
