# Supplementary material for: Molecular Insights into Fluoride Ion Uptake and Selectivity in the CLCF Fluoride/Proton Antiporter
Source: J Phys Chem B. 2025 Apr 15;129(16):4005–11. doi: 10.1021/acs.jpcb.4c08174 (PMC12035849; doi:10.1021/acs.jpcb.4c08174)
Supplement: Supplementary file 1 — jp4c08174_si_001.pdf [file jp4c08174_si_001.pdf]

Supporting Information

Molecular Insights into Fluoride Ion Uptake and Selectivity in the CLCF Fluoride/Proton Antiporter

Akihiro Y. Nakamura[1,2] and Takuya Mabuchi[2]*

1. Graduate School of Engineering, Tohoku University, 2-1-1 Katahira Aoba-ku, Sendai 980-8577, Miyagi, Japan
2. Institute of Fluid Science, Tohoku University, 2-1-1 Katahira, Aobaku, Sendai, Miyagi 980-8577, Japan

*Corresponding Author: Takuya Mabuchi (mabuchi@tohoku.ac.jp)

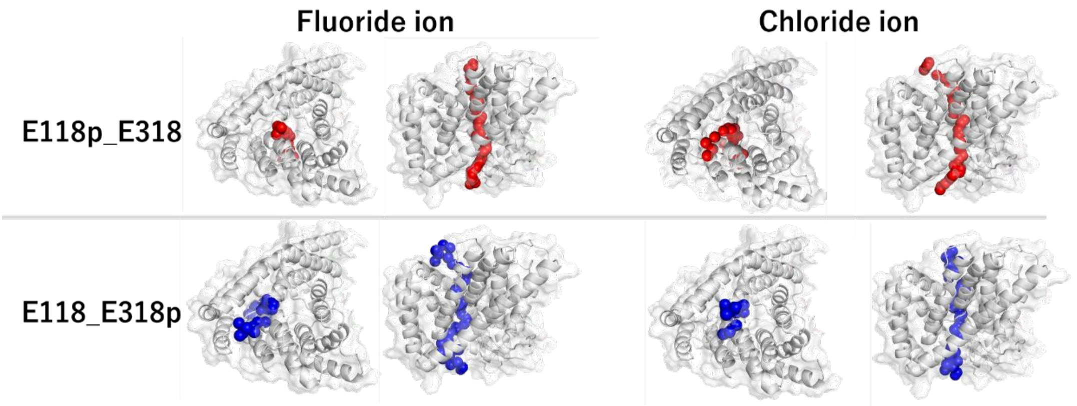

Figure S1. Snapshots of reaction coordinates of fluoride ion and chloride ion obtained from steered molecular dynamics (SMD) simulations.

S2. System equilibration with two fluoride ions at binding sites S_{cen} and S_{ext}

To examine whether two fluoride ions can remain stably bound at both binding sites simultaneously, we performed equilibration simulations under different protonation states of E118 and E318, following the original windmill mechanism.[1–3] We constructed three different systems with distinct protonation states of E118/E318: 1) protonated E118 and deprotonated E318 (E118p_E318); 2) deprotonated E118 and protonated E318 (E118_E318p); and 3) protonated E118 and protonated E318 (E118p_E318p). In each system, two fluoride ions were initially placed at the binding sites S_{cen} and S_{ext}. The equilibration procedure followed that described in the main manuscript, where all systems were equilibrated using the CHARMM-GUI protocol, followed by an NPT run for 200 ns. Our results show that in all cases, one of the fluoride ions exited the transport pathway, leaving only a single fluoride ion remaining at either S_{cen} or S_{ext} after equilibration (Figure S2). This is consistent with previous findings reported by Mills et al.[4]

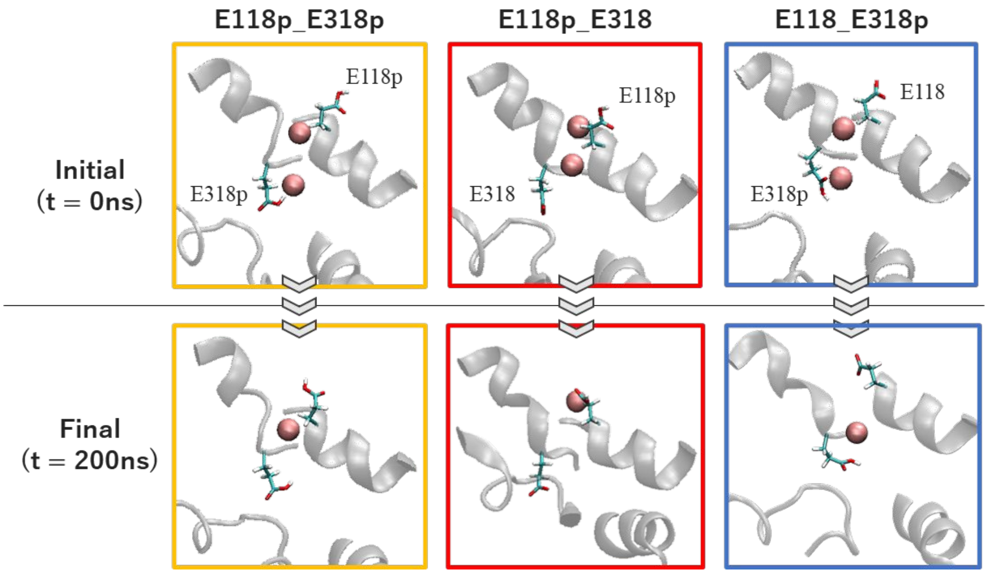

Figure S2. Snapshots of systems at initial and equilibrium states under the three different protonation states. In all cases, only a single fluoride ion remains in the protein after equilibration.

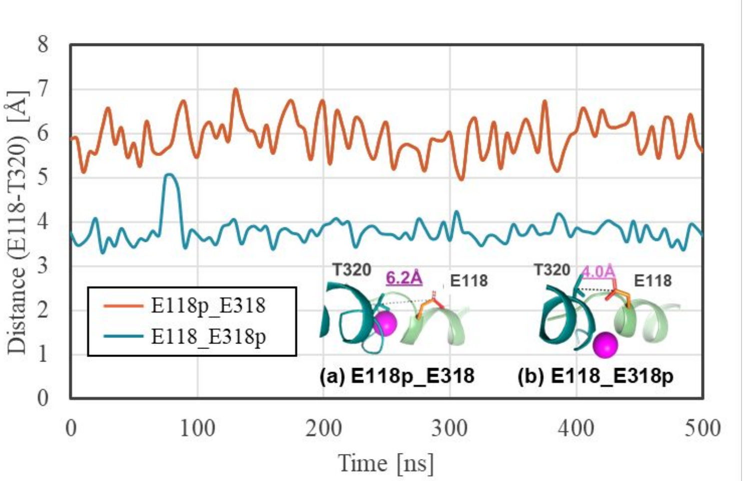

Figure S3. Distance between the center-of-mass of the side chains of E118 and T320.

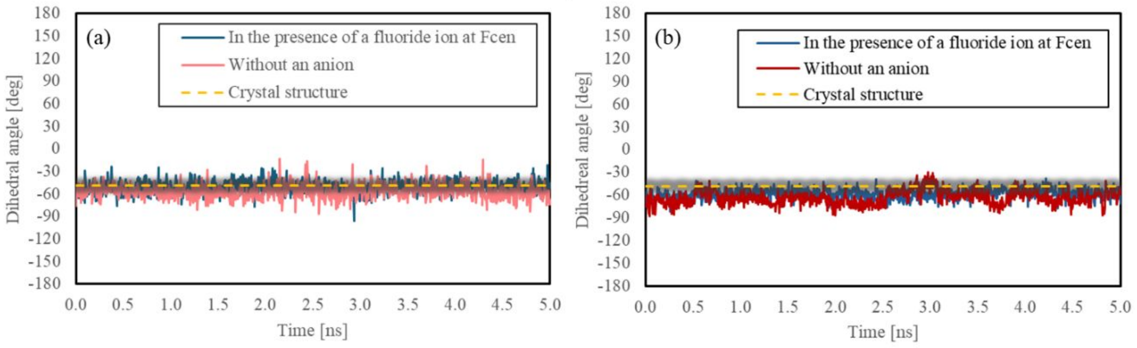

Figure S4. Time evolution of the dihedral angle of E118 for (a) E118p_E318 and (b) E118_E318p. The dihedral angles remained stable around 50 degrees, consistent with the crystal structure, indicating that E118 was always in the up position throughout the simulations.

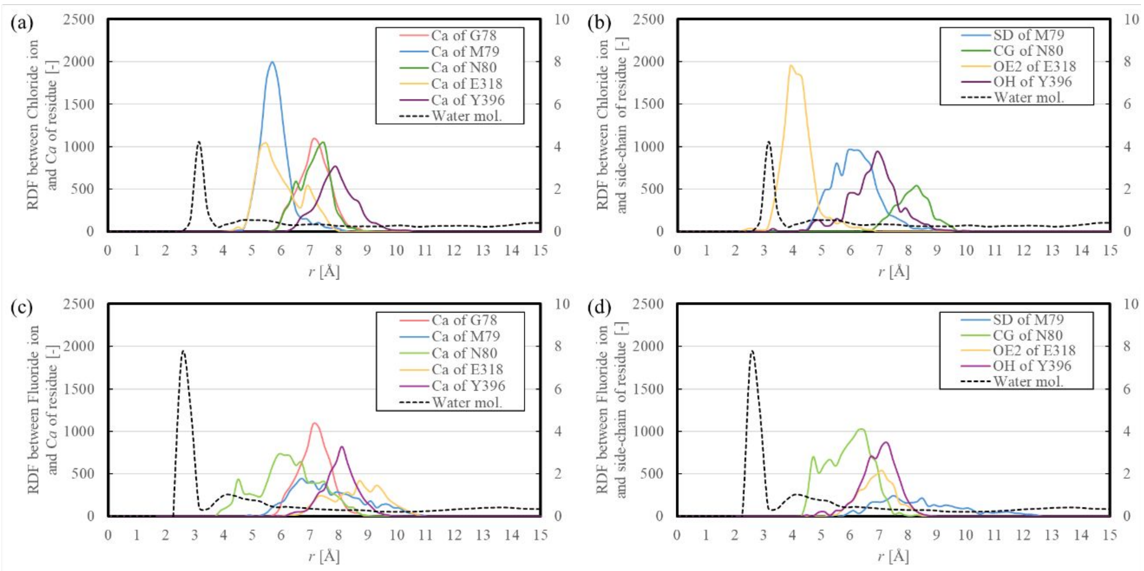

Figure S5. Radial distribution functions (RDFs) of the five closest protein residues and water molecules around anions. All cases correspond to the protonated state of E318 (E318p). (a) Backbone interactions with chloride ions, (b) Sidechain interactions with chloride ions, (c) Backbone interactions with fluoride ions, and (d) Sidechain interactions with fluoride ions. For chloride ions (a, b), the RDF between the side-chain oxygen of E318p and the chloride ion partially overlaps with the first peak of the RDF between chloride and water molecules, indicating direct binding of chloride to E318p. In contrast, the backbone of M79 exhibits a sharp peak outside the chloride solvation shell, suggesting an indirect interaction. No distinct structural features were observed for the other residues. For fluoride ions (c, d), no significant peaks were observed in either the backbone or sidechain RDFs compared to chloride, and the distribution was relatively broad. This suggests that fluoride ions do not exhibit strong specific binding to protein residues, further supporting the idea that fluoride transport occurs primarily through hydration rather than direct protein coordination.

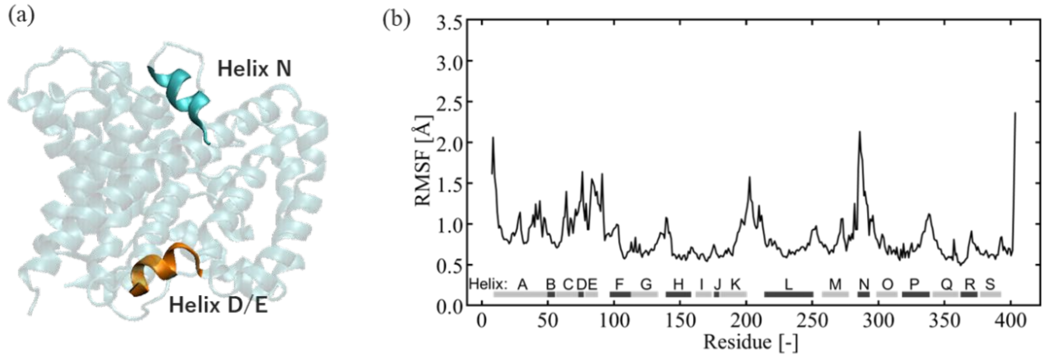

Figure S6. (a) The locations of Helix D/E and Helix N in CLCF. (b) RMSF for the Cα atoms of each residue without anions. The results indicate that Helix N exhibits similar fluctuations to those observed in Figure 4 with anions at Z_{COM} = −7.5 Å, suggesting that its structural flexibility is independent of anion presence.

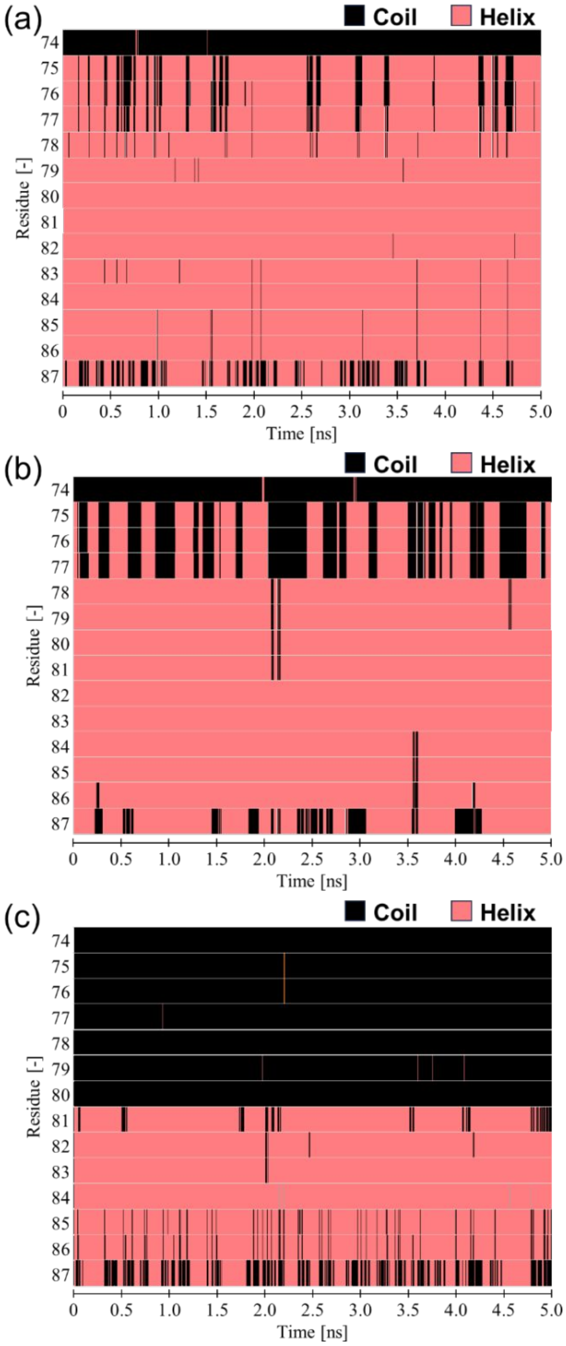

Figure S7. Time evolution of the secondary structure of amino acids in Helix D and Helix E of the CLCF F^-/H^+ antiporter from DSSP analysis.[5] (Helix D: residues 74–77, Helix E: residues 78–87). (a) Without an anion, (b) In the presence of a fluoride ion at Z_{COM} = −7.5 Å, and (c) In the presence of a chloride ion at Z_{COM} = −7.5 Å. In the absence of an anion, Helix D remains mostly intact but exhibits minor fluctuations due to its intrinsic fragility. When a fluoride ion is present at Z_{COM} = −7.5 Å, the helix fraction decreases to ~55%, indicating partial destabilization. In contrast, in the presence of a chloride ion at Z_{COM} = −7.5 Å, Helix D undergoes a helix-to-coil transition, demonstrating chloride-induced structural destabilization.

Reference

(1) Last, N. B.; Stockbridge, R. B.; Wilson, A. E.; Shane, T.; Kolmakova-Partensky, L.; Koide, A.; Koide, S.; Miller, C. A Clc-Type f-/H+ Antiporter in Ion-Swapped Conformations. *Nat Struct Mol Biol* **2018**, *25* (7), 601–606. https://doi.org/10.1038/s41594-018-0082-0.

(2) Chon, N. L.; Lin, H. Fluoride Ion Binding and Translocation in the CLCF Fluoride/Proton Antiporter: Molecular Insights from Combined Quantum-Mechanical/Molecular-Mechanical Modeling. *Journal of Physical Chemistry B* **2024**, *128* (11), 2697–2706. https://doi.org/10.1021/acs.jpcb.4c00079.

(3) Chiariello, M. G.; Alfonso-Prieto, M.; Ippoliti, E.; Fahlke, C.; Carloni, P. Mechanisms Underlying Proton Release in CLC-Type F-/H+Antiporters. *Journal of Physical Chemistry Letters* **2021**, *12* (18), 4415–4420. https://doi.org/10.1021/acs.jpclett.1c00361.

(4) Mills, K. R.; Torabifard, H. Uncovering the Mechanism of the Proton-Coupled Fluoride Transport in the CLCF Antiporter. *J Chem Inf Model* **2023**, *63* (8), 2445–2455. https://doi.org/10.1021/acs.jcim.2c01228.

(5) Kabsch, W.; Sander, C. Dictionary of Protein Secondary Structure: Pattern Recognition of Hydrogen-Bonded and Geometrical Features. *Biopolymers* **1983**, *22* (12), 2577–2637. https://doi.org/10.1002/BIP.360221211.
